# Dual-Color Bioluminescence Analysis for Quantitatively Monitoring G-Protein-Coupled Receptor and β-Arrestin Interactions

**DOI:** 10.3390/ph4030457

**Published:** 2011-02-25

**Authors:** A.K.M. Kafi, Mitsuru Hattori, Naomi Misawa, Takeaki Ozawa

**Affiliations:** 1 Department of Chemistry, School of Science, The University of Tokyo, Bunkyo-ku, Hongo, Tokyo 113, Japan; 2 PRESTO, Japan Science and Technology Agency, Tokyo, Japan

**Keywords:** GPCR, luciferase, protein interaction

## Abstract

G protein-coupled receptors (GPCRs) are crucial elements in mammalian signal transduction, and are considered to represent potent drug targets. We have previously developed a GPCR assay system in cultured cells based on complementation of split fragments of click beetle (*Pyrearinus termitilluminans*) luciferase. The interaction of GPCRs with its target, β-arrestin, resulted in strong emission of bioluminescence upon stimulation with its specific ligand. In this study, we improved precision of the GPCR assay system by using railroad worm (*Phrixothrix hirtus*) luciferase as an internal control. We generated stable cell lines harboring the railroad worm luciferase and quantitatively evaluate the extent of GPCR-β-arrestin interactions. We showed concentration-dependent bioluminescence responses for four GPCRs: β2-adrenoceptor, endothelin receptor type A, α2-adrenoceptor and human μ-opioid receptor. We also demonstrated that the variation of responses was reduced significantly by normalizing the data with bioluminescence from railroad worm luciferase. This assay system represents a simple and reliable approach for screening drug candidates in a high throughput manner.

## 1. Introduction

G-protein coupled receptors (GPCRs) form a large and diverse multigene superfamily of integral membrane proteins that are involved in many important physiological functions [1]. GPCRs control key physiological functions, such as neurotransmission, hormone and enzyme release from endocrine and exocrine glands, immune responses, cardiac and smooth muscle contraction, and blood pressure regulation. These receptors are not only crucial targets for basic biology but also prominent components for drug discovery [2,3]. A large part of efforts towards drug discovery and development is focused on finding chemicals that affect the ability of ligands to bind to GPCRs, either to inhibit or accelerate certain cellular processes. However, as our knowledge of the complexity of GPCRs' function increases, particularly when studied in a cellular environment, it has become clear that novel approaches are needed to monitor the interaction of GPCRs with their targets. β-arrestins are multifunctional adaptors that mediate the desensitization, internalization, and some signaling functions of GPCRs [3].

**Figure 1:**
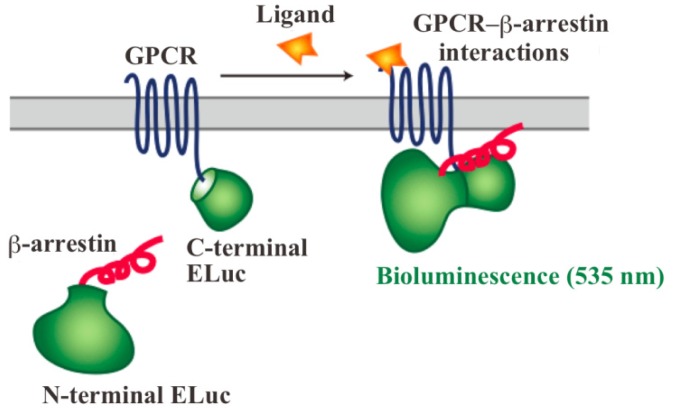
Schematic diagram showing the use of split ELuc probe to monitor the GPCR-β-arrestin interaction. The N-terminal ELuc fragment is attached to β-arrestin, and the C-terminal ELuc fragment is connected to the GPCR. Binding of a ligand to a GPCR results in its interaction with β-arrestin and then bioluminescence activity of ELuc is recovered by complementation of the ELuc fragments.

In order to assay and detect the GPCR-β-arrestin interactions, powerful technologies based on protein fragment complementation have been used for screening chemicals and drug candidates [4-8]. The principle of protein fragment complementation is that a reporter protein, which is an enzyme or a fluorescent protein, is divided into two fragments so that they cannot function alone. These fragments are fused to potentially-interacting protein partners and complementation upon interaction leads to restore its function. The restored enzyme activity or fluorescence can be detected as an indicator of protein-protein interactions [7-11]. In recent years, GFP-fragment complementation has been used for the technique of visualizing GPCR-β-arrestin interactions [12]. Localization of the interactions in living cells was visualized with the technique of GFP-fragment complementation. However, the technique has some limitations such that it often requires for high-energy excitation, which results in high background signals. In contrast, bioluminescent proteins such as firefly, *Renilla*, *Gaussia* and click beetle emit light by a biochemical reaction without any excitation sources. Taking such strong advantage of the bioluminescent enzyme, we have developed novel green luciferase fragments from click beetle (Brazilian *Pyrearinus termitilluminans*; Emerald Luc; ELuc) for the study of GPCR-β-arrestin interactions in live cells (Figure 1) [13,14]. The use of click beetle luciferase has a strong advantage of brightness; the photon count of click beetle luciferase is estimated as being over 10-fold higher than those of firefly luciferase. In addition, the spectrum of firefly luciferase is known to change in a pH-dependent manner, whereas click beetle luciferases have the property of pH independence of the spectra [15,16]. However, in spite of some advantages, the split fragments from the click beetle failed to produce sufficient levels of precision. The activity of luciferase is changed in proportion to the concentrations of d-luciferin and ATP. In addition, the number of the cells in 96-well microtiter plates is different in each well, which makes the precision worse in the data handling.

In this study, we show a method of precision-improved bioluminescence analysis for monitoring GPCR-β-arrestin interactions in living cells based on ELuc fragment complementation. To reduce fluctuation of bioluminescence between different wells, we used a red color luciferase from the railroad worm (*Phrixothrix hirtus*; RWLuc) as an internal control. The RWLuc is the best candidate as an internal control because the substrate of RWLuc is the same as that of ELuc, d-luciferin.

In addition, the emission maximum of RWLuc is 625 nm, which is distant from that of ELuc (emission maximum: 535 nm) [17,18]. We generated four kinds of stable cell lines of HEK293 cells transfected with β-arrestin (ARRB2) and different GPCRs, which were connected with a pair of ELuc fragments. Moreover, RWLuc was transfected into the HEK293 cells, and then new stable cell lines were generated. To evaluate quantitatively the effect of chemicals that act on GPCRs, bioluminescence intensity from the ELuc was divided by that from RWLuc. By this procedure, standard deviations of the bioluminescence responses were improved and quantitative evaluation became possible on 96-well microtiter plate formats.

## 2. Experimental

### 2.1. Materials

The cDNA of click beetle (Brazilian *Pyrearinus termitilluminans*) luciferase (Emerald Luc; ELuc) and Emerald Luciferase Assay Reagent were obtained from Toyobo Co. Ltd. (Japan). The cDNA of railroad worm (*Phrixotrix hirtus*) luciferase (RWLuc) was a gift from Dr. Y. Ohmiya (AIST, Japan). The human cDNAs of α2-adrenoceptor (ADRA2A; NM_000681.3) and μ-opioid receptor (OPRM1; NM_001008503.1) were obtained from GeneCopoeia Inc. (Rockville, MD). The cDNA library of human brain, DNA polymerase and restriction, modification enzymes were obtained from Takara Bio Inc. (Japan). Damgo, endothelin 1 (ET-1), and epinephrine were purchased from Wako Pure Chemical Industries Ltd. (Japan). DRAQ5™ dye was obtained from Biostatus Ltd. (Leicestershire, UK). Mouse anti-V5 antibody and donkey anti-mouse IgG antibody tagged with AlexaFluoro 488, Hoechst 33342, and expression vectors of pcDNA3.1/myc-His(B) and pcDNA4/V5-His(B) were purchased from Invitrogen Corp. (Carlsbad, CA).

### 2.2. Construction of Plasmids

**Figure 2:**
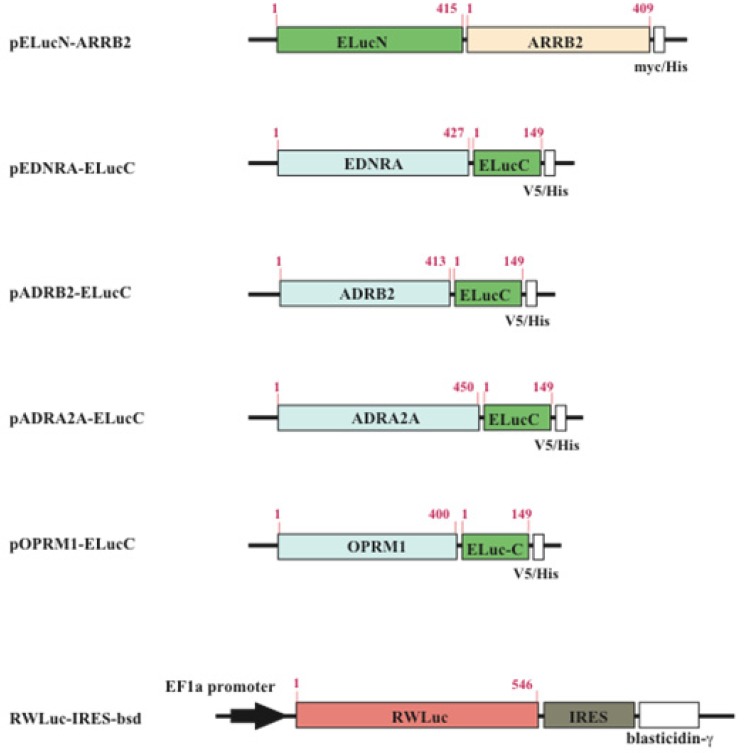
Schematic structures of the cDNA constructs. ELucN, N-terminal fragment of the luciferase; ELucC, C-terminal fragment of the luciferase; ARRB2, β-arrestin; EDNRA, Endothelin receptor A; ADRB2, β2-adrenoceptor; OPRM1, human μ-opioid receptor; ADRA2A, α2-adrenoceptor; RWLuc, Red color luciferase. The numbers indicate the amino acid positions.

The cDNAs used for the plasmid construction were generated using the standard polymerase chain reaction (PCR) with gene specific primer and Pyrobest DNA polymerase. The cDNAs of β-arrestin (ARRB2; NM_004313.3), β2-adrenoceptor (ADRB2; NM_000024.5) and endothelin receptor type A (EDNRA; NM_001957.3) were amplified from the human brain cDNA library. The PCR fragments were subcloned into restriction enzyme sites of mammalian expression vectors, pcDNA3.1/myc-His(B) or pcDNA4/V5-His(B) (Figure 2). All PCR fragments were sequenced using a genetic analyzer (ABI310; Applied Biosystems). For transfection of the cells, the expression vectors were purified using a DNA purification system (Purelink HiPure Plasmid Filter, Invitrogen Corp.).

### 2.3. Transfection, Cell Culture and Generation of Stable Cell Lines

The HEK293 cells were cultured in Dulbecco's modified Eagle's medium (DMEM, high glucose) supplemented with 10% fetal bovine serum (FBS), 100 unit/mL penicillin, and 100 μg/mL streptomycin at 37 °C in an incubator with 5% CO_2_. Transfection of plasmids into the cells was performed with a reagent (TransIT Transfection; TransIT^®^-LT1; Mirus Co., TX). In order to obtain cell lines which stably expressed GPCR and β-arrestin, the transfected cells were screened with both 0.8 mg/mL geneticin (G418) and 0.04 mg/mL zeocin. To generate stable cell lines which expressed RWLuc, the HEK293 cells that have already expressed the GPCR and β-arrestin were further transfected with the plasmid of RWLuc, and then the cells were screened with 0.05 mg/mL blasticidin. The expression of proteins in each stable cell line was confirmed by the existence of both red and green bioluminescence.

### 2.4. Measurement of Bioluminescence Spectra

The stable cell lines were cultured on 96-well microtiter plates and transferred into a small vial, measurement of spectrum, for 1 min for each sample. Where necessary, the cells are stimulated with 1× 10^−6^ M isoproterenol for 12 min. Bioluminescence spectra were measured using an AB-1850 LumiFL-spectrocapture (ATTO Corp., Tokyo, Japan). Bioluminescence spectra were collected for 1 min in each sample.

### 2.5. Bioluminescence Assays of GPCR-β-Arrestin Interactions

The cells were cultured with a phenol red free DMEM supplemented with 1% FBS on 96-well microtiter plates, and incubated at 37 °C in 5% CO_2_ overnight. The cells were stimulated with different concentrations of ligands and incubated for a given time at 37 °C in 5% CO_2_. Before measurement of luciferase activities, 100 μL/well of the Emerald Luciferase Assay reagent (Toyobo Co. Ltd., Japan) was added in each well. The ELuc and RWLuc activities were measured using a 96-well microtitier plate reader (TriStar LB941; Berthold Technologies GmbH and Co. KG, Germany) with one band-pass filter of 525 ± 25 nm for bioluminescence from ELuc and the other band-pass filter of 630 ± 30 nm for bioluminescence from RWLuc. All measurements were performed five or six times with different wells of culture plates. The values were averaged and presented with standard deviations.

## 3. Results and Discussion

### 3.1. Generation and Characterization of Cell Lines Stably Expressing RWLuc

The dissecting sites of ELuc are very important to obtain maximum responses and to avoid background signals. We have previously identified new dissecting sites from the ELuc with a significant improvement of the signal-to-background ratio and absolute photon counts [14]. Based on the previous study, the combination of N-terminal ELuc (1-415 aa) and C-terminal ELuc (394-542 aa) has been applied to monitoring the interaction between GPCRs and β-arrestin (Figure 1). Among various kinds of GPCRs, β2-adrenoceptor (ADRB2) was chosen for the initial experiments. The ADRB2 was connected with the C-terminal ELuc fragments, whereas β-arrestin was connected with the N-terminal one. Both fusion proteins were stably expressed in the HEK293 cells, named HEK293-ARRB2-ADRB2 cells. The use of HEK293 cells has a few advantages; it is possible to express exogenous proteins in higher level and growth rate of the cells is relatively fast. The cells were seeded on 96-well microtiter plates and then stimulated with the ligand, isopreternol. Bioluminescence increased concomitantly with increasing concentrations of isopreterenol and the maximum responses obtained was 15–40-fold higher than the background bioluminescence (data not shown). However, absolute photon counts of the bioluminescence changed in each well of the microtiter plates because of a little difference in d-luciferin, ATP and the number of cells. In order to minimize such unwanted fluctuation, we further constructed a cell line that stably expressed RWLuc in the HEK293-ARRB2-ADRB2 cell line. Of more than 100 subcloned cells, we obtained a cell line that stably expressed the RWLuc, which was named HEK293-ARRB2-ADRB2-RWLuc.

**Figure 3:**
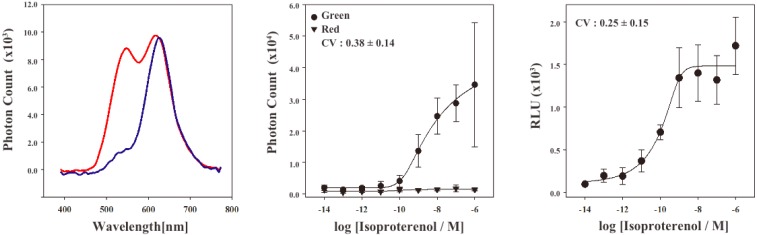
(**a**) Bioluminescence spectra of HEK293-ARRB2-ADRB2-RWLuc cells in the presence (red line) and the absence (blue line) of isoproterenol. The transfected cells were cultured on 96-well microtiter plates, transferred into small vial, and then bioluminescence spectra were measured; (**b**) Concentration dependence of isoproterenol on the photon count of bioluminescence. The bioluminescence through the filter (525 ± 25 nm) is shown in black circle (green color bioluminescence) and that through the filter (630 ± 30 nm) was in black triangle (red color bioluminescence). Photon counts were taken for 2 s/well using a microplate reader and the mean luminescence intensities and their standard deviation were determined at each ligand concentration (n = 5). Inset data is the value of co-efficient variation; (**c**) Changes of relative luminescence unit (RLU) at various concentrations of isoproterenol; The RLU values were calculated from the raw data of bioluminescence in (**b**). Inset data is the value of co-efficient variation.

In order to characterize the stable cell line, the bioluminescence emission spectra were examined using a lumino-spectral meter (Figure 3A). The bioluminescence spectrum from the cell line in the absence of ligands showed a single emission peak at the wavelength of 625 nm. The emission maximum is consistent with that of RWLuc, indicating that the RWLuc was stably expressed in the cells. In the presence of 1.0 μM isoproterenol, we observed an enhanced emission peak at the wavelength of 535 nm, attributed to complementation of split ELuc fragments. Absolute photon count at 535 nm from ELuc was almost the same as that at the 625 nm from RWLuc, indicating that RWLuc is useful for internal control of ELuc fragment complementation.

Next, to examine characteristics of the newly generated HEK293-ARRB2-ADRB2-RWLuc stable cell line, we investigated the quantitative assessment of the isoproterenol-induced ADRB2–β-arrestin interaction in the cells. The HEK293-ARRB2-ADRB2-RWLuc stable cells were grown on 96-well microtiter plates, and then stimulated with each concentration of isoproterenol for 12 min. The bioluminescence through a band-pass filter of 525 ± 25 nm was separated from the bioluminescence through a band-pass filter of 630 ± 30 nm. Figure 3B shows raw data of bioluminescence through the filter of 525 ± 25 nm with different concentrations of isoproterenol. The photon counts of bioluminescence increased concomitantly with increasing concentrations of isoproterenol from 1.0 × 10^−9^ to 1.0 × 10^−5^ M. On the other hand, a little change in the photon counts from bioluminescence through 630 ± 30 nm was found in the various concentrations of isoproterenol. Background luminescence in the absence of isoproterenol was 924 (525 ± 25 nm) and 123 (630 ± 30 nm) and mechanical noise of plate readers in the absence of cells was 16 (525 ± 25 nm) and 11 (630 ± 30 nm). The maximum photon counts produced were about 35-fold higher than the background luminescence. Absolute photon counts through 630 ± 30 nm were lower than those through 525 ± 25 nm although the maximal response was obtained at 625 nm in the spectral analysis (Figure 3a). This may be due to the fact that the sensitivity of the plate reader over 600 nm was lower than that of the spectrometer and, therefore, the photon counts through 525 ± 25 nm became higher than those through 630 ± 30 nm.

To evaluate quantitatively the effect of isoproterenol, bioluminescence intensities from ELuc were normalized against those from RWLuc. The normalization was performed such that the bioluminescence through the filter of 525 ± 25 nm was divided by the bioluminescence through the filter of 630 ± 30 nm, which was defined as a relative luminescence unit (RLU). Figure 3C shows the bioluminescence changes in the RLU, which was evaluated from photon counts in the presence of different concentrations of isoproterenol (Figure 3b). The RLU values reached a maximum in the presence of 1 × 10^−6^ M isoproterenol, which was 13-fold higher than that in the absence of isoproterenol. The EC_50_ value obtained for isoproterenol was 1.6 × 10^−10^ M. The value was lower than that reported previously by the β-galactosidase fragment complementation assay (2.7 × 10^−8^ M) [4].

To compare the standard deviations between the raw data of bioluminescence intensity and its normalized values of RLU, coefficient of variation (CV) was calculated. The CV values were 0.38 ± 0.14 for the raw data of bioluminescence and 0.25 ± 0.15 for the RLU. This result demonstrated that the standard deviation of the bioluminescence responses was significantly improved by normalization with the bioluminescence of RWLuc.

**Figure 4:**
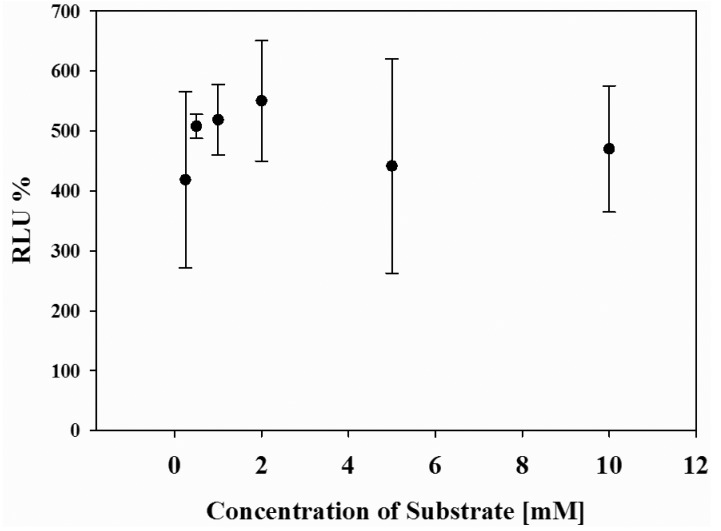
Effect of d-luciferin concentrations on the relative luminescence unit (RLU). The HEK293-ARRB2-ADRB2-RWLuc stable cells were grown on 96-well microtiter plates and stimulated with 1.0 × 10^−6^ M isoproterenol. Bioluminescence intensities through each filter (525 ± 25 nm and 630 ± 30 nm) were obtained and the relative luminescence unit was calculated. The standard deviations were determined at each d-luciferin concentrations (n = 5).

Finally, the effect of cell number in wells and concentrations of the substrate on the luminescence response of the system was systematically investigated. Upon 80–90% cell populations, no difference in the maximal response was obtained (data not shown). However, when cells were confluent about 100% of well, the photon counts decreased because of the cell death. In addition, when concentration of d-luciferin was changed from 0.5 mM to 10 mM, the maximum photon counts were almost the same (Figure 4), indicating that the present dual-color assay is not influenced by cell population or substrate concentrations.

### 3.2. Application to Different GPCRs

**Figure 5:**
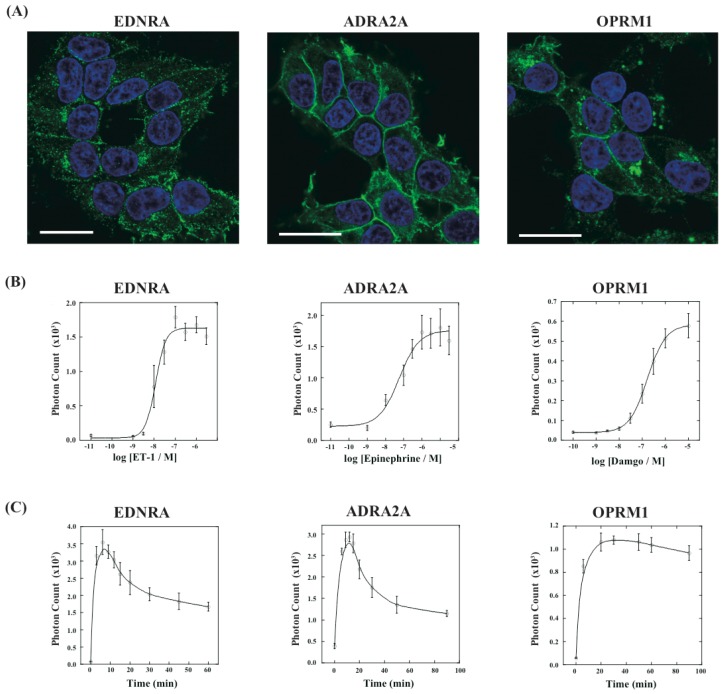
Characterization of cell lines stably expressing GPCRs and β-arrestin. (**A**) Localizations of endothelin receptor type A (EDNRA), α2-adrenoceptor (ADRA2A) and human μ-opioid receptor (OPRM1) were shown in green. The GPCRs were recognized by anti-V5 antibody and visualized using AlexaFluoro 488. The nuclei were stained with Hoechst 33342 (blue). Overlaid images are shown. Bar: 20 μm; (**B**) Dose-response curves for each ligand. The cells were cultured on 96-well microtiter plates and stimulated for 10 min. The mean luminescence intensities were determined at each ligand concentration (*n* = 6); (**C**) Time course analysis of GPCR-β-arrestin interactions. The stably expressed cells in each well were treated with 1.0 × 10^−7^ M endothelin-1 for EDNRA, 1.0 × 10^−6^ M epinephrine for ADRA2A, and 1.0 × 10^−6^ M damgo for OPRM1. The luciferase activities were measured in the subsets of cells (*n* = 6).

Next, we have applied this assay system to different kinds of GPCRs. We have constructed three new cell lines that stably expressed GPCRs of endothelin receptor type A (EDNRA), α2-adrenoceptor (ADRA2A) and human μ-opioid receptor (OPRM1), each of which was connected with the C-terminal fragment of ELuc. The cell lines were named HEK293-ARRB2-EDNRA, HEK293-ARRB2-ADRA2A and HEK293-ARRB2-OPRM1, respectively. The localization of each receptor was examined by immunostaining with Alexa-Fluoro 488 under a fluorescence microscope. All GPCRs localized on the plasma membrane although some portions of the receptors existed in the endoplasmic reticulum (Figure 5A). We next examined responses of bioluminescence for each specific ligand, endothelin-1 for EDNRA, epinephrine for ADRA2A and damgo for OPRM1. The bioluminescence responses depended on the concentration of each ligand and the maximum responses were 15-30-fold higher than the background bioluminescence (Figure 5B). Interestingly, time-dependant analysis of photon counts upon ligand stimulation exhibited different response time for the bioluminescence (Figure 5C). In the case of EDNRA, the maximum bioluminescence reached at 5–7 min after addition of endothelin-1. Decreases in the bioluminescence after maximum response were swift, and the response at 60 min was almost half of the maximum response. On the other hand, time-dependent responses of the bioluminescence for ADRA2A and OPRM1 were slower than the response for EDNRA. The maximum responses for ADRA2A and OPRM1 were obtained at 10 min and 20 min after stimulation of the corresponding ligands, respectively. The differences of the time-dependent changes in the bioluminescence between GPCRs were also observed in the previous study, which might be originated from dissociation of GPCR-β-arrestin interactions and/or decomposition of GPCRs [14].

**Figure 6:**
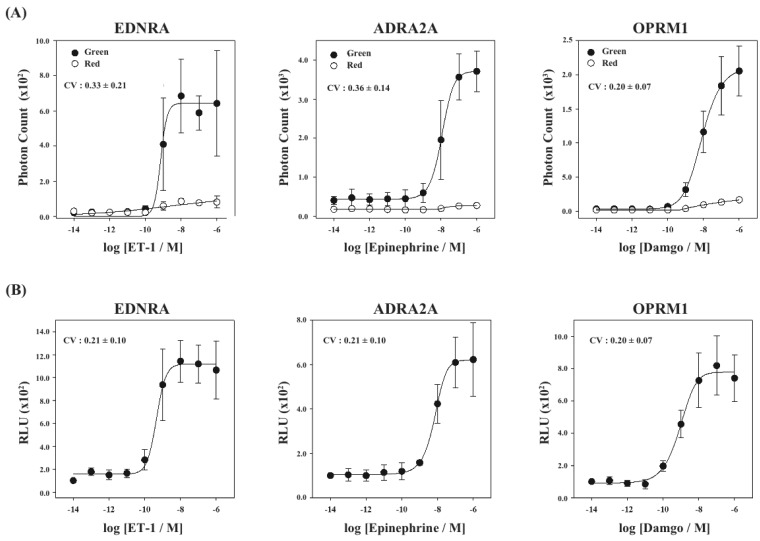
Concentration dependences of ligands on EDNRA-β-arrestin, ADRA2A-β-arrestin and OPRM1-β-arrestin interactions. (**A**) Raw data of bioluminescence. The bioluminescence through the filter (525 ± 25 nm) was shown in black circle and that through the filter (630 ± 30 nm) was in open circle. Photon counts were taken for 2 s/well using a microplate reader and the mean luminescence intensities and their standard deviation were determined at each ligand concentration (n = 5). Inset data is the value of co-efficient variation; (**B**) Changes of relative luminescence unit (RLU) at various concentrations of each ligand. The RLU values were calculated from the raw data of bioluminescence in (**A**). Inset data are the values of co-efficient variation.

Finally, using the cell lines of HEK293-ARRB2-EDNRA, HEK293-ARRB2-ADRA2A and HEK293-ARRB2-OPRM1, we generated stable cell lines which stably expressed RWLuc, named HEK293-ARRB2-EDNRA-RWLuc, HEK293-ARRB2-ADRA2A-RWLuc and HEK293-ARRB2-OPRM1-RWLuc, respectively. The bioluminescence intensities with the filter of 525 ± 25 nm increased with increasing in each ligand, and the maximum responses were approximately 25-fold for EDNRA, 15-fold for ADRA2A and 30-fold for OPRM1 higher than the background bioluminescence (Figure 6A).

On the other hand, no remarkable change in the bioluminescence through the filter of 630 ± 30 nm was obtained. When we calculate RLU, we found increases in the RLU values; 10-fold for endothelin receptor type A (EDNRA), 6-fold for α2-adrenoceptor (ADRA2A) and 8-fold for human μ-opioid receptor (OPRM1), respectively (Figure 6B). The EC_50_ values were 6.9 × 10^−9^ M for EDNRA, 3.4 × 10^−10^ M for ADRA2A and 1.0 × 10^−9^ M for OPRM1. The CV values calculated from the raw data of bioluminescence and those of RLU were 0.33 ± 0.21 and 0.21 ± 0.10 for EDNRA, 0.37 ± 0.14 and 0.21 ± 0.10 for ADRA2A, and 0.20 ± 0.07 and 0.20 ± 0.07 for OPRM1, respectively. In any cases, standard deviations were even or improved by introducing the process of normalization of the raw data with RWLuc.

The normalized curves show different maximal signal amplitudes (RLU) from one GPCR to another. One of the reasons is the difference of maximal bioluminescence responses for the GPCRs in raw data: When the higher maximal response of photon counts was obtained after ligand stimulation, the maximal RLU value became higher number. The other reason is the differences of expression levels of RWLuc. We could not control the expression level or RWLuc or GPCRs between the four kinds of stable cell lines, which resulted in the differences of the RLU values. These facts indicate that it is impossible to compare RLU values between the different GPCRs.

A more widely used technology is the bioluminescence resonance energy transfer (BRET) assay, of which basic concept is identical to the present method. However, because of spectral overlaps between bioluminescence and fluorescence, rigorous data analyses are needed to distinguish non-specific interaction from true oligomeric protein interactions. In comparison to such existing method, the present method presents strong advantages in terms of the detection period—less than 10 min, a high signal-to-background ratio of the bioluminescence, and high sensitivity of absolute photon counts.

Dual color assay reporters are also commonly used to improve experimental accuracy and precision [19]. The activity of one reporter gene generally represents molecular events in a target cell, while the activity of the second reporter gene provides an internal control. Thus, dual reporter assays often allow for more reliable interpretation of the experimental data by reducing extraneous influences. Dual-reporter applications utilizing firefly luciferase in combination with either chloramphenicol acetyltransferase (CAT), β-galactosidase (β-Gal) or β-glucuronidase (GUS) have been used [20,21]. However, those co-reporter combinations diminish advantages of luciferase. For example, while the luciferase assay can be performed and quantitated in seconds, the CAT, β-Gal and GUS assays are endpoint assays requiring lengthy incubation periods prior to quantitation. In addition, these other reporters are limited in their sensitivity and in the range of their linear response; care must be taken not to exceed these ranges. In this context, a GPCR assay method with dual-luciferases has recently been developed with a built-in control using *Renilla* luciferase [22]. Normalization of firefly luciferase activity with *Renilla* luciferase activity assists in compensating for potential variability in cell density and nonspecific effects caused by the compounds. However, the reporter assay system is not so accurate because the *Renilla* luciferase activity has to be measured separately with a different substrate, coelenterazine. This assay system can be affected from the differences in the d-luciferin and ATP concentrations. In comparison to such previous methods, the present assay system provides simple assay procedure and allows us to monitor the effects of ligands on GPCR quantitatively. There is no need to consider unwanted differences in d-luciferin and ATP concentrations inside the cells, which is unique and quite useful for constructing a high-throughput screening system.

In the design of this assay system, the GPCRs were connected with C-terminal fragment of ELuc. Effects of the fusion construct on the original function of GPCRs were not evaluated extensively in the present study. However, the C-terminal fragment of ELuc is composed of only 149 amino acids, which is much smaller than the β-galactosidase fragment and GFP. In the previous studies, it has been reported that GPCRs fused with GFP or β-galactosidase fragment bound to their agonists and antagonists in a normal concentration ranges and their internalization events resulted [4,12]. Considering these data, there may be no effect of connection of the ELuc fragment with GPCR on their specific functions.

## 4. Conclusions

In this work, we have applied split fragments from click beetle luciferase to monitoring new GPCR-β-arrestin interactions. To justify variables of data originated from the cell number, ATP concentrations, the amount of d-luciferin and transfection efficiency, we generated the stable cell lines that expressed full length of RWLuc as an internal control. We normalized the obtained data with the bioluminescence of RWLuc, and precision of the normalized values was significantly improved in comparison to simple evaluation from absolute photon counts. Usefulness of this new system was demonstrated with four GPCRs; β2-adrenoceptor, endothelin receptor type A, α2-adrenoceptor and human μ-opioid receptor, and the present method will be further applicable for any kinds of GPCRs if their interacting proteins are present. Because the assay procedure is quite simple and the obtained data is reliable, the methods become a powerful new approach for the study of GPCR analysis and for screening of drug candidates.
